# Factors influencing fathers’ involvement in the care of hospitalized preterm newborns in Balaka, Malawi

**DOI:** 10.1186/s12887-023-04253-1

**Published:** 2023-08-29

**Authors:** Patani Mhango, Alinane Linda Nyondo-Mipando

**Affiliations:** 1 Department of Health Systems and Policy, School of Global and Public Health, Kamuzu University of Health Sciences (KUHeS), Private Bag 360, Blantyre, Malawi; 2 Centre for Reproductive Health, Kamuzu University of Health Sciences (KUHeS), Private Bag 360, Blantyre, Malawi; 3 Maternal and Fetal Health Group, Malawi Liverpool Wellcome Programme, Blantyre, Malawi

**Keywords:** Preterm newborn care, Fathers' involvement, Barriers, Facilitators

## Abstract

**Background:**

Malawi has one of the highest incidences of premature birth, with twice the mortality compared to full-term. Excluding fathers from preterm newborn care has negative consequences, including father feeling powerless, missed bonding opportunities with the newborn, additional strain on the mother, and negative family dynamics such as breakdown in communication, reduced trust, and strained relationships. In Malawi, there is no deliberate policy to have fathers involved in preterm care despite having high incidence of preterm birth and neonatal mortality. There is also limited literature on the factors that influence fathers’ involvement in the care. The aim of the study was to explore factors influencing fathers’ involvement in the care of hospitalized preterm newborns.

**Methods:**

A descriptive qualitative study design was used, guided by Theory of planned behaviour and the model proposed by Lamb on male involvement. Sixteen in-depth interviews were conducted with fathers of preterm infants purposively and conveniently sampled in June 2021. Interviews were digitally recorded and transcribed verbatim. Data were organized and analyzed using Nvivo software and thematic analysis approach was used because the approach allows deeper understanding of the data, identification of patterns and themes, and provides rich insights into participants’ experiences and perspectives.

**Results:**

The barriers and facilitators that influence a father’s involvement in the care of preterm newborn babies include: perceived difficulty with care activities and benefits of involvement, gender roles and socio-cultural beliefs, work and other family responsibilities, social support, baby’s physical appearance/nature and health status, feedback from the baby, multiple births, and hospital’s physical environment and provision of basic needs.

**Conclusion:**

The study found that fathers value their involvement in caring for hospitalized preterm newborns but face barriers. Evidence-based interventions like education programs, training sessions, and support groups can help fathers overcome barriers and promote better outcomes for infants and families.

## Background

The first 28 days of life (newborn period) are the most critical for a child’s survival. In 2020, 2.4 million newborns died, which accounts for 47% of all under-five deaths [1]. A majority (43%) of these deaths occurred in Sub-Saharan Africa where neonatal mortality is 27 deaths per 1000 live births [1]. Preterm birth is one of the dominant risks in neonatal mortality [2], and it accounted for 35% of neonatal deaths worldwide in 2019 [3]. Malawi has one of the highest preterm births and neonatal mortality. Preterm birth is estimated to range from 7.9 to 29.7% [4], and neonatal mortality rate is estimated at 27 deaths per 1,000 live births [5]. Neonatal mortality rate is higher in Malawi when compared against neighboring countries, with Zambia reporting 24 deaths, and Tanzania had 20 deaths in 2021 [6]. The Malawi government developed guidelines and protocols for preterm newborn care in order to reduce neonatal mortality rates. However, Malawi still lacks a deliberate policy on male involvement in the care of preterm newborns, despite the Ministry of Health recognizing the importance of involving fathers.

Fathers’ involvement in the care of preterm newborns is critical to influencing the newborns’ behavioural and psychological outcomes, [7] as well as overall health [8, 9]. These positive outcomes are because fathers (or male partners) play a vital role in making decisions about the health of the children in the home [8]. Fathers who are involved in care activities become good parents [9], finding fatherhood more fulfilling and considering themselves naturally important to their children [10]. Fathers that are involved in care are more likely to have a positive feeling about their interactions with the children [11, 12], better understand, and appreciate their children and develop a rich bond with them. Therefore, fathers’ engagement in care practices needs to be initiated from the first days of the child’s life, even in situations when the mother is recovering and able to provide care [13].

Male involvement in the care of preterm newborns can have several benefits, that could help reduce the high mortality. When fathers share caregiving responsibilities, it can lessen the burden on mothers and prevent the mothers from feeling overwhelmed [14, 15]. Moreover, fathers who participate in the care of their preterm newborns are more likely to be well-informed about their baby’s health, adhere to medical advice, and actively contribute to decision-making about their child’s care. These factors can ultimately lead to better health outcomes for the baby. Lamb et al. argue that male involvement has three components, which include availability (accessibility), engagement, and responsibility [10]. Accessibility is where a father is physically present but only plays a passive or supervisory role to ensure the child is safe. Engagement represents the more intensive, one-on-one interaction with the child, while responsibility is the ability of the father to know what is needed so that they can plan and arrange for the provision of certain aspects of child care [10].

Just like the mothers, the birth of a preterm baby in a family is equally stressful for the fathers. Fathers need targeted nursing interventions that are different from those applied to mothers [16]. Factors influencing their involvement in care need to be explored because fathering a preterm newborn is multifaceted and predisposed by personal differences, and traditional, and contextual factors that would help develop interventions that respond to fathers’ needs. In Malawi, there is no policy on male involvement in the care of preterm newborns despite the numerous benefits that come with fathers’ involvement. Furthermore, there is no literature on male involvement in preterm infant care despite Malawi having a high incidence of preterm births and high neonatal mortality. As such, exploring the factors that influence fathers’ involvement in the care of their hospitalized preterm newborns would be key for the health care providers to professionally involve fathers in the daily care activities and build a strong foundation for continued involvement even after discharge from the hospital.

There are both barriers and facilitators to fathers’ involvement in the care of preterm newborns. Barriers can result in few men reported to be actively involved. Some of the barriers are social-cultural beliefs that influence gender norms [17, 18], the baby’s physical nature [7, 13, 19–21], the hospital environment [13, 19, 22], work and other family responsibilities [19–21, 23], and exclusion by health care staff [24]. On the other hand, factors that facilitate fathers’ involvement in care are good communication from health care providers [7, 22], social support [19, 24, 25], encouragement from male champions in the community [18, 26] and their spouses [19, 24, 27], feedback from the infant itself when held by the father [24, 28], and multiple births [19].

The study utilized the constructs of the Theory of Planned Behaviour to assess fathers’ involvement based on the components of male involvement as proposed by Lamb et al. [10] (availability, engagement, and responsibility). The Theory of Planned behaviour explains what encourages a person to exhibit a specific kind of behaviour [29], in light of the several social factors outside one’s control that may encourage or impede that particular behaviour. The theory proposes that behaviour is not only born out of an intention but also out of one’s aptitude to exhibit that behaviour [30]. According to the theory, attitudes, subjective norms, and perceived behavioural control are factors that influence the intention to perform a behaviour [31]. This means an individual is likely to have an intention to perform certain behaviours such as caring for their preterm newborns if they have a positive attitude, believe that social pressure allows the performance of that behaviour and that they believe they have what it takes to perform those behaviours correctly [29, 32]. If one has these three, they are likely to have a stronger intention and more likely to perform the behaviour. However, several external factors may encourage or discourage an individual performance of that particular behaviour, despite the intention being there [32]. The aim of the study was to explore factors influencing fathers’ involvement in the care of hospitalized preterm newborns.

## Methodology

### Study design

A descriptive qualitative research design was used in this study. In this qualitative research, the researcher seeks to understand the participants’ lived experiences in a manner that would help conceptualize the experiences and increase the researcher’s knowledge and understanding of human experiences [33].

### Study setting

The study was conducted in Balaka district, Malawi. Balaka is a rural district in the southern region bordering Mangochi, Neno, Machinga, and Ntcheu districts. It has a population of 438, 379 [34]. As of 2017, the district had an estimated 91,176 women of childbearing age with approximately 18,592 annual expected pregnancies and deliveries [35]. The study was conducted at Balaka District Hospital in June 2021 where all preterm newborns in the district are referred. The district was selected due to its high infant mortality rate estimated at 51 per 1,000 live births, which is above the national average of 42 per 1,000 live births as stated in the 2017 Demographic and Health Survey [36].

### Sample size

The sample consisted of sixteen fathers who had preterm neonates hospitalized at Balaka district hospital. The number resonates with methodological literature on saturation of qualitative data [37, 38], which argues that saturation could be reached with between 9 and 24 interviews, especially in research like this where the sample is relatively homogeneous and the research is aims-focused [38]. Data were collected until saturation was reached.

### Sampling

The participants were chosen by convenient and purposeful sampling to get rich data. The maximum variation sampling technique was used to gain greater insights from the participants. Maximum variation was achieved by recruiting participants of different ages, several children, religions, levels of education, occupation, and ethnic background. Eligible fathers were identified by a member of the clinical staff and referred to the research assistant who obtained permission from them to take part in the study.

### Data collection

Data were collected in June 2021 using in-depth interviews (IDIs) conducted by an experienced and trained research assistant, who has a Public Health background. The research assistant training covered objectives of the study, data collection procedures, qualitative data collection, ethics, and communication skills. The training was conducted to ensure that research assistant understands the importance of ethical considerations and is able to conduct research in a responsible and ethical manner. Interviews were conducted in a private room at the hospital and the privacy allowed participants to get engaged in a comfortable conversation making it easy for them to share experiences as compared to them having to fill out a survey questionnaire [39]. The interviews were conducted in the language of participants’ choice (Chichewa or English) using a semi-structured guide and were audio-recorded. Each took a maximum of 40 min.

The main questions of the interview were:

1. What activities do you do with your child when you come to visit?
2. In those activities, you are yet to do; do you intend to try to do them?
3. How do you feel about the activities you are involved in as you provide care to the baby?
4. How capable do you think you are in providing the care needed for your preterm baby?
5. How do people that are considered important to you react, as you get involved in the provision of care to your preterm baby?
6. What factors enable you to be involved in the care of your preterm newborn?
7. What factors make it difficult or impossible for you to be involved in the care of your preterm newborn?

### Trustworthiness of the study

To ensure the trustworthiness of the study, the researcher put into serious consideration the validity, dependability, and conformability of the results. To ensure validity, the researcher employed procedures that ensured the credibility and transferability of the data. To ensure credibility, the researcher ensured the data collection tools were checked and vetted by an expert in health-related qualitative research. The tools were also pilot-tested to see if they were gathering the data they were supposed to. During the interviews, rapport was built from the beginning to ensure participants were fully engaged and probes were used to ensure the respondent adequately addresses the questions. Adequate time was allocated to analysis to ensure interpretations are in line with the data from the participants. Transferability was accomplished by ensuring participants had varied demographic characteristics so that there was wide representation and provides a detailed description of context, data collection, and analysis approaches used to allow replication in a different place and different populations [40].

The dependability of the research process was ascertained by the clear description of procedures and processes involved during the research. These included objective recruitment of study participants and a proper description of data analysis methods employed to allow others who may wish to audit and replicate them to do so [41]. Conformability was achieved by ensuring results are objective and neutral, independent of the researcher’s views [40]. Conformability enabled the findings to be consistent with those from similar related studies. Personal beliefs and opinions did not affect the research findings.

### Data analysis

Data were managed using NVivo version 12. Data collection and analysis were done concurrently. Audio-recorded interviews were transcribed verbatim in Microsoft Word immediately after completion of the interviews. Rigorous review and quality control were performed by the authors to ensure accuracy and consistency between the transcripts and audio recordings. The analysis was conducted by the authors using qualitative thematic analysis approach [42]. The authors familiarized themselves with the data by reading and re-reading the transcripts while taking notes of initial codes as they read the transcripts. The coding scheme combining a priori codes and data-driven codes was developed based on key concepts from the Theory of Planned Behaviour and Lamb’s model of fathers’ involvement, and repeated reading of the transcripts. This approach allowed for the incorporation of existing theoretical constructs while also allowing new themes to emerge. Codes were then grouped into categories which combined similar codes and these later were organized under overarching themes based on recurring patterns, concepts, or ideas in the data and data reduction techniques were applied to identify the most illustrative examples within each themes while preserving the core meanings and experiences expressed by the participants. The authors then examined the relationships between themes, identify connections, and explore the implications of the findings to develop a coherent and comprehensive description. They also paid attention to avoid overwrapping themes and those with very minimal data to stand alone as a theme. These were combined with other themes to better illustrate the findings. The data analysis process involved iterative revisits to previous steps to achieve a better representation of the findings and encompassed multiple discussions of the results between the authors.

### Ethics

The study was approved by the Malawi College of Medicine Research Ethics Committee (COMREC Protocol # P.11/20/3208). To protect study participants’ privacy and confidentiality, a unique personal identification number was assigned to each individual in the study. This number was used instead of their names on the data collection forms (DCFs) and within the study databases. The only identifiable information about the participants was contained in the study’s Informed Consent Forms, which were securely stored in locked filing cabinets at the Kamuzu University of Health Sciences. Access to these cabinets is restricted solely to the researchers involved in the study. All study interviews were performed in private rooms to protect participant confidentiality. Permission was also granted by the Director of Health and Social Services for Balaka district.

## Results

### Participant demographics

The study had 16 participants from different demographics as per Table 1 below. Participants belonging to Lomwe and Ngoni tribes were the majority with 44% and 38% respectively. In terms of age, 38% were those aged 25–29 and 31% were those 35 years and above. The mean and median age were 29.8 years and 29 years respectively with the standard deviation 5.57 years. The majority of the participants were first-time fathers (44%) and three quarters were Christians. Slightly more than half of the participants (56%) were educated to the secondary school level and majority (63%) were not employed.

**Table 1:** Participant characteristics

**Variable**	**Attribute**	**No.**	**%**
Tribe	Chewa	1	6
	Yao	2	13
	Ngoni	6	38
	Lomwe	7	44
Age	20–24	3	19
	25–29	6	38
	30–34	2	13
	35 and above	5	31
Number of children	1	7	44
	2	1	6
	3	4	25
	4 and above	4	25
Religion	Christian	12	75
	Non-Christian	4	25
Education level	Primary	5	31
	Secondary	9	56
	Tertiary	2	13
Employment status	Formal Employment	3	19
	Self employed	3	19
	Not employed	10	63

### Factors influencing fathers’ involvement

There were five main categories of factors that influence fathers’ involvement in the care of preterm newborns. These are personal factors, interpersonal factors, infant factors, environmental factors, and economic factors as in Table 2 below.

**Table 2:** Factors influencing fathers’ involvement

**Themes**	**Sub-themes**
Fathers’ personal motivational factors	Perceived difficulty with care activities
	Perceived benefits of involvement
Interpersonal factors	Gender roles and socio-cultural beliefs
	Work and other family responsibilities
	Social support
Infant factors	Baby’s physical appearance/nature
	Baby’s health status
	Feedback from the baby
	Multiple births
Environmental factors	Hospital’s physical environment
Economic factors	Provision of basic needs

### Fathers’ personal motivational factors

Fathers reported that their perception of the difficulty of activities they are involved in and the perceived benefit of being engaged in those activities influenced their involvement in care.

#### Perceived difficulty of activities

The fathers described that their perception of the difficulty of the tasks could not stop them from being involved in the care of the newborns and nothing was difficult for them to do. The fathers reported that despite some care activities such as bathing the infant and skin-to-skin care are perceived as difficult when provided to a preterm newborn, it did not stop them from taking part in those activities.

*“When I was told by the nurse to put the baby in a kangaroo position, I did not find it difficult to do. It is like allowing the baby to sleep on my chest and that was not a problem.” *(IDI 14).

#### Perceived benefits of involvement

Involvement in the care of the preterm infants was enabled by the fathers’ belief that it was important for them to be involved because it provided them with an opportunity to show their love to the babies and enhanced the bonding.

*“The child gets to know his father because the closeness to the child gives her the familiarity of what his father is. Even if she grows and starts recognizing things she will know that this one is my father and be able to differentiate who her father is from the other men who might wish to carry her.” (IDI 9).*

Furthermore, it also helped the mothers realise that the fathers do care for the babies despite them being born preterm

*“It is important because my wife will also be happy that the kids are loved by their father and it makes me very happy because this is something treasured.” *(IDI 15).

### Interpersonal factors

The fathers perceived that several interpersonal factors affected their level of involvement in the care of their preterm newborns. These factors are gender roles and cultural beliefs, work and family responsibilities, provider attitude, social support, and sharing experiences with fathers who have had preterm newborns.

#### Gender roles and cultural beliefs

Unfriendly gender roles and cultural beliefs prevented the fathers from being involved in the care of the babies. The fathers considered some roles as belonging to women and they could only do them when the mother is sick or busy with other tasks. Fathers’ inability to be involved in the roles is based on the belief that infant care was traditionally a female role while the fathers have to provide for the family’s needs

*“That (caring for infants) is part of the mother’s care. For us men, it is to search and provide for them.” *(IDI 8).

*“When the mother is busy with something, I can do like changing his diaper, but in case she is not busy, Aaah [laughing] some things are for ladies.”* (IDI 11).

#### Work and family responsibilities

Various responsibilities from work and family acted as barriers to involvement in the care of preterm newborns since fathers spent their time at work and attending to other family responsibilities such as caring for the children left at home at the time the mother was in the hospital with the newborn. These multiple responsibilities reduced the time fathers could spend at the hospital and caring for the newborns.

*“I need to provide for them and need to work to get food that I was told I should be giving the mother of the child, so my concern is there on time.”* (IDI 14).

*“I stay with my wife and the children, and I have to make sure that I care for the children since those who go to school have to go and I have to come here.”* (IDI 6)

#### Provider’s attitude

The fathers acknowledged that the support they get from the health care providers has been a facilitator to their involvement in the care. The fathers reported that providers had been so supportive and communicative about the condition of the baby and how best the parents can provide the needed care.

*“The nurses are very good because they are always telling us what we need to do in the care of the baby and everything they are telling us here seems to be working.”* (IDI 7).

On the other hand, the behavior of other providers prevented the fathers from being involved. The fathers reported that some health providers were rude in communicating with them while other providers preferred communicating with the mothers than the fathers which impeded fathers’ involvement in care.

*“This other nurse is always shouting at us and you sometimes get afraid that she will shout at you when you touch the baby.” *(IDI 6).

#### Social support

The social support the fathers receive from their friends and relatives was also reported as one factor that enables them to get more involved in the provision of care. The fathers got encouragement from their social networks telling them that they need to focus on providing the care the baby needs because they were not the first to have preterm newborns.

*“They are helping me a lot because they even give me encouraging examples of people who are still alive and strong who were born preterm. That encourages me much that I have to provide good care to this child.” *(IDI 12).

Some of the family and friends do come to visit them at the hospital or call them to check how they are doing with the baby.

*“They do call me to hear how they are faring as well as encourage me to be checking on the wife and the child regularly.” *(IDI 6).

Talking to other men who have gone through the same situation was also considered a facilitator to involvement. It was reported that these had firsthand experience on how to handle preterm newborns and how their involvement helped the babies grow. Talking to such men acted as motivation to get more involved in the provision of care.

*“These people have passed through this already so their words are of great importance and we follow since we know that it is coming from someone who has experienced what he is saying.” *(IDI 2).

Furthermore, one participant reported that he is involved in the care so that he could be a role model to other men, that the mother needs to be supported in the care of the babies.

*“What makes me happy in doing this is that I want other fathers to take it as a lesson that we need to be always available to our children because caring for the children is for both father and mother*.” (IDI 9).

### Infant factors

Fathers’ involvement in care was also influenced by infant factors. These range from the babies’ physical appearance and nature, the health status of the infant, feedback from the baby, and multiple births.

#### Baby’s physical appearance or nature

The physical nature of the baby prevented fathers from getting more involved in the care. The fathers were afraid because the baby was fragile-looking and too small and thought they will harm the baby when they try to get involved in the activities.

*“I might not know how to touch or hold him since there might be ways how to touch such a child when bathing him. The child is too small so it might be difficult to ably do it*.” (IDI 12).

#### Baby’s health status

The health status of the baby was another infant factor that influences fathers’ involvement in care. Fathers found it difficult to get involved when the baby was sick or not in good health. However, the positive changes they noticed acted as an encouragement for them to get more involved. Others had seen their babies transferred from the isolette to an open crib, and were able to finally hold them.

*“The improvement encourages me because it shows a difference from the time the child was born to now so when I look at those positive changes it encourages me that my child will be like me one day a fully grown person so I have to do more for him.” *(IDI 7).

Furthermore, babies’ growth helped remove the fears that they might lose the baby. Dealing with these fears helped them accept the situation and motivated them to get more involved in the care.

*“It is encouraging to me that the child is growing fast and is healthy which motivates me to do more.” *(IDI 5).

#### Feedback from the baby

The fathers described that the feedback they get from the babies while providing care acted as a facilitator to their involvement. They reported that the positive feedback given by the babies such as stretching and smiling made the fathers feel acknowledged and made them want to do more with and for the babies.

*“The first day I carried the child, she was not comfortable or open up as how a child is supposed to do when the child is born. However, for the past three days when I carry the child, I see that the child tends to stretch its arms, smile, and do other things. So, this tends to make me happy and encouraged.” *(IDI 14).

#### Multiple births

All the fathers who had twins acknowledged that having two babies was an enabler for them to get involved because twin birth made it difficult for the mother to handle both babies alone. Inability to handle both babies at the same time allowed fathers to come in and help. For example, when the mother is feeding or holding one baby, the fathers attend to the other baby.

*“When they were giving milk to one I had to carry the other one who was also waiting to be given after the other one. So I just carried one and after giving the other one milk I then carried the other one.” *(IDI 1).

### Environmental factors

The physical environment in the hospital is another factor that affected involvement. Fathers reported that they were unable to take on some tasks because the ward is just an open space where they could not privately take on some roles such as skin-to-skin care. After all, they were in sight both men and women in the ward.

*“The problem is that you find that in the ward the baby is in, there are several women and each can see what every other person is doing. As such, it becomes difficult to help with other things such as kangaroo care.” *(IDI 8).

Furthermore, in situations where the newborn was in the isolette, the fathers were unable to interact with the baby because there was limitation on who is allowed to touch the baby. Care guidelines allow only the mother and health care provider to touch the baby in the isolette.

*“She is in the incubator so I cannot even touch her.” *(IDI 9).

### Economic factors

Fathers acknowledged that their inability to raise enough funds for the provision of care affects their involvement in the care. They reported that all that the baby needs at the moment requires money which most of them do not have. As such, they spent much of their time away from the hospital searching for resources that would enable them to provide for the baby’s needs, which ultimately reduced the time the fathers would be at the facility providing care to the baby.

*“The most challenging thing is money because I may not manage since the work I do is not that so much paying considering it is in the village and what I get there may not be enough to provide for the baby’s needs.” *(IDI 12).

In addition, the advent of COVID-19 has worsened the situation on the fathers’ ability to generate income and provide the basic needs for the babies.

*“Due to the COVID-19 pandemic, a lot of things have changed that even the way income is being generated these days is a bit a challenge so this might be the thing that may hinder the work of the provision of care that involves the provision of all the necessity a child needs and the availability in the life of this child.” *(IDI 2).

On the other hand, fathers that were formally employed and had a steady source of income reported that being financially stable helps them spend more time at the facility with their babies and gives them more opportunities to get involved in the provision of care.

*“Having enough money to provide for my child would help me be at home most of the time and spend more time with the mother and the child.” *(IDI 7).

## Discussion

The findings from the current study suggest that several factors act as barriers as well as facilitators to fathers’ involvement in the care of hospitalized preterm newborns. Guided by the Theory of Planned Behaviour and Lamb’s model of fathers’ involvement, it is evident that fathers’ personal motivational factors, interpersonal factors, infant factors, environmental factors, and economic factors affect fathers’ involvement in care. The findings suggest that fathers did not find tasks such as skin-to-skin care difficult and believed their involvement was an opportunity to express love to the newborn and develop a bond with the child. These findings are consistent with other studies that reported that fathers considered child care provision as responsibility for both the father and the mother, and that fathers’ involvement enhances the bonding between the father and the baby [43]. This suggests that more involvement in caregiving would be a motivating factor for fathers to frequently visit the baby at the hospital and create more chances for them to get involved in care as it would help them feel more responsible for their newborns.

Interpersonal factors such as unfriendly gender roles and cultural beliefs made fathers consider some roles as feminine hence not taking part in care. Earlier studies [44–46] found that childcare is culturally considered a feminine role. This necessitates a shift of mentality to help fathers realise that they can also take part in caregiving activities. Work and other responsibilities have been reported in the current study as impeding fathers’ involvement in the care of hospitalized preterm newborns. This is consistent with findings from other studies [19, 47–50], which reported that multiple responsibilities prevented the fathers from being engaged in caregiving activities. It is worth noting that at the time of the study, the Malawi labour laws were not allowing for paternal leave for those formally employed, and also the majority of the participants were not in formal employment which meant they still needed to work to earn something for the families.

Fathers felt the communication and support from the health providers helped them get better engaged in care. These findings are similar to other studies which acknowledged that inclusive interaction about childcare with the providers helped the fathers feel recognized and get more involved in the care of their preterm newborns [13, 43, 51]. These findings, however, differ from other studies [7, 52] where fathers felt sidelined by the health providers in the provision of care to the newborns which mean providers’ attributes and approach to work may affect fathers’ involvement in care. Additionally, the findings show that getting help from different individuals, sharing experiences, or talking to fellow fathers who are having or have ever had preterm babies helped them cope with the situation and encouraged them to get more involved in care activities. The findings are consistent with those from other studies conducted in Malawi where parents received social support from family and community members [44, 53]. The experiences shared with fellow fathers of preterm newborns helped fathers deal with feelings of being isolated [54], and helped them realise that they are not alone [55]. These findings imply that talking to fellow fathers of preterm newborns or being able to interact with those that have ever gone through a similar situation helps the fathers get to learn from each other on how best to handle the situation; hence the need to provide safe spaces where they could be having such interaction. A case could also be made from this on how important male champions could be in reproductive health.

Infant factors such as physical appearance, health status, and feedback from the baby influence fathers’ involvement in care. The findings from the current study suggest that physical appearance and fragile-looking skin impeded fathers’ involvement since they feared they could harm the baby. These findings are similar to another study conducted in Malawi, which reported that caregivers including fathers, were afraid of holding the preterm newborns because they looked small and fragile, and the caregivers feared harming them [44], which is consistent with findings from studies conducted in other countries [51, 56]. This fear made fathers unwilling to touch and hold the babies [7, 19, 20, 57]. On the other hand, fathers felt encouraged to get more involved when they heard and saw that the babies’ health status was improving. This was further enhanced by the feedback they were getting from the baby whenever they hold or talk to the baby. These findings are similar to other studies where fathers reported feeling encouraged by babies’ developmental improvements [58], and the feedback helped them deal with their fears and get more involved in care [19, 20, 59]. On the other hand, the absence of improvement in health status and a lack of feedback from the newborn may lead to withdrawal from care by the father [19]. Providers supporting fathers in the hospitals, therefore, have a duty to help fathers understand the different non-verbal cues the baby makes so that lack of them may not act as a deterrent from involvement in care.

The presence of multiple births had a positive influence on the fathers’ involvement in the care of preterm newborns because fathers felt the mother on her own was lacking since there were two babies to be attended to and the fathers had to come in to help with caregiving activities. These findings are consistent with a Swedish study which found that twin birth meant there was a need for more people to help with the caregiving activities and enabled the fathers to get more involved [19]. For instance, when the mother was done breastfeeding one, the father took the responsibility of burping that one while the mother breastfed the other twin.

The physical environment at the hospitals where the babies were admitted was reported as another factor impeding the fathers from being involved in care activities. Fathers in the current study needed more private space and not an open ward where everyone could see them. Other studies have also reported that lack of privacy in open-spaced hospital wards prevented the fathers from getting involved in care [60]. However, these findings do not support previous research, which found that the open-spaced beds allowed the fathers an opportunity to see their colleagues hold the babies and get involved in care activities which helped them realise that it was possible to get involved in care [19]. As such, there is a need for the healthcare staff attending to the preterm newborns to frequently engage fathers and get their preferences, and if resources are available, provide more privacy to those that may need it so that they get more engaged in care.

Economic factors influence fathers’ involvement in care. As per the model of male involvement proposed by Lamb et al. [10], responsibility entails the father being able to provide for what the newborn may need. The current study suggests that fathers that had formal employment considered their financial stability as a facilitator to involvement in care while those financially unstable would be spending more time running errands and doing piece works to earn some money to provide for the baby thereby limiting the time they could take part in care activities. These findings are consistent with other studies, which reported that the ability to provide for basic needs meant the father had time to be involved in care [23, 61] and further reduced parents’ psychological distress which usually affects their involvement in care activities [61]. In Malawi, it was reported that due to financial challenges, fathers were unable to provide for what is needed for the baby [26, 44].

Some of the limitations of the study are the fathers were reporting about their own experiences thereby making it prone to self-reporting bias, and interviews were conducted by a female research assistant which may have prevented the fathers from further opening up as compared to if the interviewer was male. The study was also conducted during the time Coronavirus disease (COVID-19) was at its peak which resulted in there being limited visitations to the health facilities as a preventive measure and affected recruitment of participants. Despite these limitations, the participants in the study were from varied cultural, ethnic, educational, and economic backgrounds which allowed for varied experiences in preterm care. Furthermore, the concurrent data collection and analysis helped determine saturation and also allowed the researcher to get in-depth data from the participants especially when it was noticed certain data gaps in the preliminary analysis. The use of the theory of Planned Behaviour and the Model of fathers’ involvement as engagement, accessibility, and responsibility helped the study to get the experiences that were more focused and also measure the chances of the fathers having the intention to be involved in care.

## Conclusions

The study findings suggest that several factors impede or facilitate fathers’ level of involvement in care. These factors range from those considered personal such as perceived benefit to involvement, those related to the newborn, financial, facility environment, gender roles, sociocultural norms, support from health providers and relatives, and other responsibilities the fathers have. As such, health care providers and policymakers have a role to play in ensuring more fathers are taking part in the care of their preterm newborns since its impact is well known to surpass the cost. Fathers could be supported through education programs, training sessions, and support groups, which can help fathers overcome barriers and promote better outcomes for preterm newborns and families. Education programs can provide fathers with knowledge and skills related to neonatal care and the benefits of their involvement. Training sessions can help fathers develop practical skills and receive feedback from healthcare providers. Support groups can provide emotional support, a sense of community, and opportunities to share experiences and learn from other fathers. These interventions can also help promote gender equality and shared responsibility for caregiving within families.

## Data Availability

The datasets used in the study are available from the corresponding author on request.

## Abbreviations

COMREC: College of Medicine Research Ethics Committee
COVID-19: Coronavirus disease
DCF: Data Collection Forms
IDI: In-depth Interviews
KUHeS: Kamuzu University of Health Sciences
MNCH: Maternal, Neonatal and Child Health
YO: Years Old
